# Coherently Tunable Triangular Trefoil Phaseonium Metamaterial

**DOI:** 10.1038/srep21083

**Published:** 2016-02-16

**Authors:** D. M. Nguyen, Cesare Soci, C. H. Raymond Ooi

**Affiliations:** 1 Centre for disruptive photonic technologies, Nanyang Technological University, Singapore, 637371; 2 CINTRA CNRS/NTU/Thales, UMI 3288, Research Techno Plaza, Singapore 637553; 3 Department of Physics, University of Malaya, 50603 Kuala Lumpur, Malaysia

## Abstract

Phaseonium is a three-level Λ quantum system, in which a coherent microwave and an optical control (pump) beams can be used to actively modulate the dielectric response. Here we propose a new metamaterial structure comprising of a periodic array of triangular phaseonium metamolecules arranged as a trefoil. We present a computational study of the spatial distribution of magnetic and electric fields of the probe light and the corresponding transmission and reflection, for various parameters of the optical and microwave beams. For specific values of the probing frequencies and control fields, the phaseonium can display either metallic or dielectric optical response. We find that, in the metallic regime, the phaseonium metamaterial structure supports extremely large transmission, with optical amplification at large enough intensity of the microwave thanks to strong surface plasmon coupling; while, in the dielectric regime without microwave excitation, the transmission bandwidth can be tuned by varying the control beam intensity. Implementation of such phaseonium metamaterial structure in solid-state systems, such as patterned crystals doped with rare-earth elements or dielectric matrices embedded with quantum dots, could enable a new class of actively tunable quantum metamaterials.

Metamaterials are artificially engineered photonic structures displaying special optical properties, such as negative refractive index^1^,^2^ which are not found in the nature^3^. Over the last decade, metamaterials have been realized for a number of photonic applications, including cloaking^4^ and sub-diffraction-limited imaging devices^5^,^6^. Tunable metamaterials, where the ensemble or individual response of metamolecules can be varied by external stimuli, such as optical or magnetic fields, or mechanical deformations, are key to the development of a new generation of metadevices with optical response “on demand”^7^. “Coherent control” of the optical properties of metamaterials is an emerging area of research: quantum coherent effects were recently exploited in photonic crystals with controllable band pass^8^,^9^, lasing without inversion^10^,^11^,^12^,^13^, enhanced propagation of surface plasmon polaritons^14^, and modulation of coherent perfect absorption^15^,^16^.

Laser-controlled quantum media in which the optical properties can be tailored by coherent excitations may be realized out of phaseonium. The term phaseonium refers to quantum coherent materials comprising of three level, atomic-like systems, where electromagnetic transparency can be induced by a control laser^11^. In a three-level Λ scheme, optical properties of the phaseonium change as the result of quantum interference, when a laser field drives a transition^8^,^9^. In this work, we propose a new metamaterial structure made of a periodic array of triangular phaseonium metamolecules arranged as a trefoil that can display interesting optical properties including coupling of localized surface plasmon polaritons and gain.

Two different coherent light sources, a coherent optical light (laser) and a coherent microwave light (maser), are used to control the optical properties of the phaseonium metamolecules, while a probe light with different frequency is tuned to map its spectrum. The tuning parameters are the control laser and maser Rabi frequencies and the incident probe angle. We show that, independently of the tuning parameters, the phaseonium metamaterial displays both metallic (negative real part of the permittivity) and dielectric (positive real part of the permittivity) behavior at different probe frequencies. In the metallic regime, the excitation of localized surface plasmon polaritons at the sharp edges of the corrugated structure leads to a unique field distribution with nearly vanishing transmission. Only around the phase matching frequency, where plasmons coupled between different metamolecules are excited, light transmission becomes possible. In the dielectric regime, light is strongly localized in the structure, particularly its magnetic field component; trapping slows light down, and the real part of the effective refractive index increases significantly. For large microwave intensities, the dielectric phaseonium metamaterial acquires unusual characteristic of a metal with gain, amplifying both transmission and reflection of the probe. This may enable unique applications in controllable optical/plasmonic devices for telecommunication and optical data processing.

## Phaseonium Metamaterial Structure

**Figure 1:**
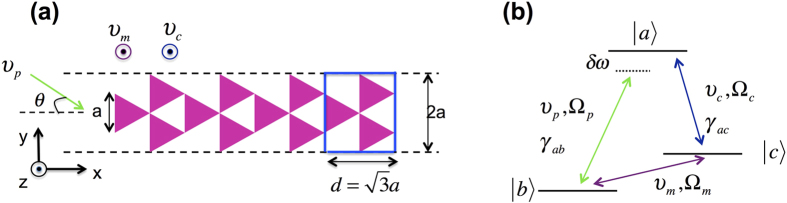
Schematic of (**a**) the triangular trefoil metamaterial structure and (**b**) the three-level system quantum phaseonium. Microwave and control light beams with frequencies *v*_*m*_ and *v*_*c*_ illuminate the structure in the *z*-direction. The probe beam illuminates the structure in the *x* − *y* plane with incidence angle *θ* with respect to the *x*-axis. Periodic boundaries surround the structure in the *y*-direction (dashed lines). Probe light is set at transverse electric (TE) or transverse magnetic (TM) and polarization. The TE light has the electric field component in the *z* direction, out of the *x* − *y* plane while the electric field vector of the TM wave is in the *y* direction.

Figure 1a shows the metamaterial structure composed of periodic array of triangular metamolecules made up of quantum coherence material (phaseonium). The unit cell of the periodic structure consists of three triangular metamolecules of edge *a*, arranged in a symmetrical trefoil as indicated by the blue rectangular box in Fig. 1a. Each unit cell measures 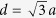 in the *x*-direction and 2*a* in the *y*-direction. In our simulations, the unit cell is repeated four times in the *x*-direction and periodic boundary conditions are assumed in the *y*-direction. The scaling relationship between the size of the unit cell and the incident probe wavelength, *λ*, is given by *a* = 2*λ*/3. This asymmetric structure is designed to support light trapping and guiding within the periodic array, while minimizing reflection when light propagates from left to right. In the regime where phaseonium is dielectric, the trefoil metamaterial behaves as a dielectric waveguide. Multiple reflections and interference between metamolecules limit transmission bandwidth, so that the spectral width of transmission window can be tuned (from narrow to broad) simply by adjusting the control beam intensity. Moreover, the sharp edges of triangular metamolecules induce strong plasmonic field enhancement and strong surface plasmon coupling, which enhance light transmission when phaseonium is set to the metallic state.

As shown in Fig. 1a, the microwave and control beams are assumed to illuminate the metamaterial structure in the *z*-direction (perpendicular to the plane of the metamolecules), while the probe light illuminates the structure in the *x*−*y* plane, with an incident angle *θ* with respect to the *x*-axis. We treat the phaseonium as a three-level quantum system in Λ configuration, as shown in Fig. 1b, where *ħω*_*ac*_ and *ħω*_*ab*_ are the energy difference between levels 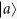 and 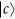 and levels 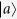 and 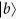 in the stationary state, respectively. The 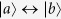 transition is probed by a laser with frequency *v*_*p*_ and electric dipole Rabi frequency Ω_*p*_. The 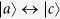 and 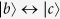 transitions are controlled by an external control laser and a maser with frequencies *v*_*c*_ (in the optical domain) and *v*_*m*_ (in the microwave domain) and Rabi frequencies Ω_*c*_ and Ω_*m*_, respectively. The control beam is on resonance for a stationary atom with 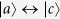 transition *ω*_*ac*_ = *v*_*c*_, while the probe is detuned by *δω* = *ω*_*ab*_ − *v*_*p*_ around stationary resonance frequency *ω*_*ab*_ of the 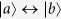 transition.

**Figure 2:**
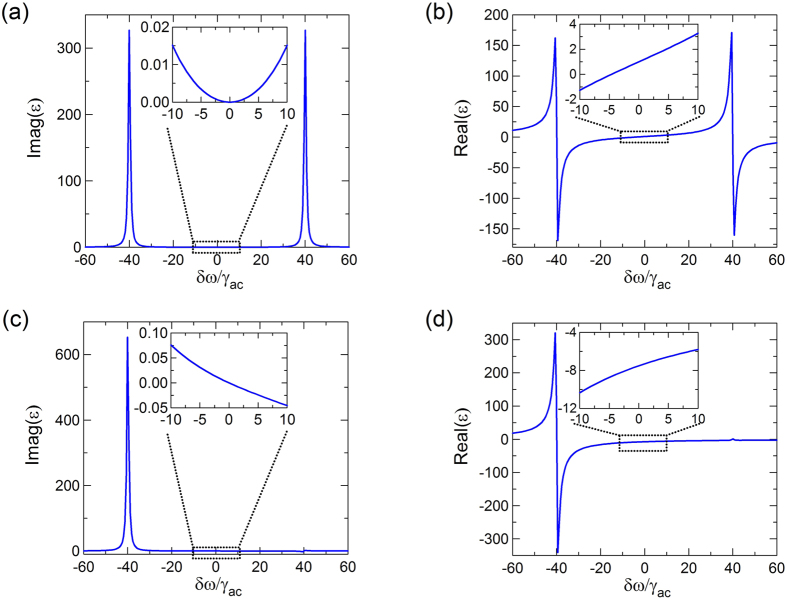
Optical permittivity of phaseonium with and without microwave excitation: (**a**) Absorption (imaginary part of the permittivity) and (**b**) dispersion (real part of the permittivity) without microwave excitation (Ω_*m*_/*γ*_*ac*_ = 0); (**c**) absorption and (**d**) dispersion with microwave excitation (Ω_*m*_/*γ*_*ac*_ = 0.1).

The permittivity of phaseonium can be tuned by varying the Rabi frequencies of the 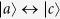 transition driven by the control laser, and of the 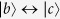 transition driven by the maser^9^. Figure 2 shows an example of the dependence of the phaseonium permittivity profile on the microwave beam intensity (see Method for calculation details). In this example, the phaseonium parameters are set as Ω_*c*_/*γ*_*ac*_ = 40 with decay rate *γ*_*ac*_ = *γ*_*ab*_ = 10^7^ s^−1^, and we consider two cases: without microwave excitation (Ω_*m*_/*γ*_*ac*_ = 0), and with microwave excitation (Ω_*m*_/*γ*_*ac*_ = 0.1). Without microwave excitation (Fig. 2a,b), the system shows the conventional EIT dispersive peaks for zero detuning of control laser^11^. The imaginary part of the permittivity shows two peaks at *δω*/*γ*_*ac*_ = ±40 (Fig. 2a) and is always positive, while the real part of the permittivity (insets of Fig. 2b) is positive for 40 > *δω*/*γ*_*ac*_ > −5 (dielectric regime) and negative for −40 < *δω*/*γ*_*ac*_ < −5 (metallic regime). When the microwave excitation is turned on (Fig. 2c,d), the EIT profile is distorted. For large Ω_*m*_ (in our case Ω_*m*_/*γ*_*ac*_ = 0.1) the system shows gain when *δω*/*γ*_*ac*_ > 0, the real part of the permittivity is negative for broad range −40 < *δω*/*γ*_*ac*_ < 40, while the imaginary part of the permittivity become negative near *δω* = 0 (see inset of Fig. 2c). The physical effect of the microwave beam is to reduce the permittivity peak at positive detuning and to increase the permittivity peak at negative detuning (compare Fig. 2a–d); overall this reduces the value of the real part of the permittivity, allowing it to acquire negative values near *δω* = 0. By this mechanism, the microwave beam can also tune the optical response of phaseonium from dielectric-like (positive real part of the permittivity) to metallic-like (negative real part of the permittivity).

## Results and Discussions

We firstly focus on the basic scheme in which the microwave beam is absent to investigate EIT of the probe field for different strengths of the control beam. We investigate the transmission *T* and reflection *R* spectra of the structure and the corresponding spatial distribution of the electric and magnetic fields with various input conditions. Since without microwave irradiation the imaginary part of the phaseonium refractive index is always positive, the metamaterial is expected to behave as a lossy waveguide.

**Figure 3:**
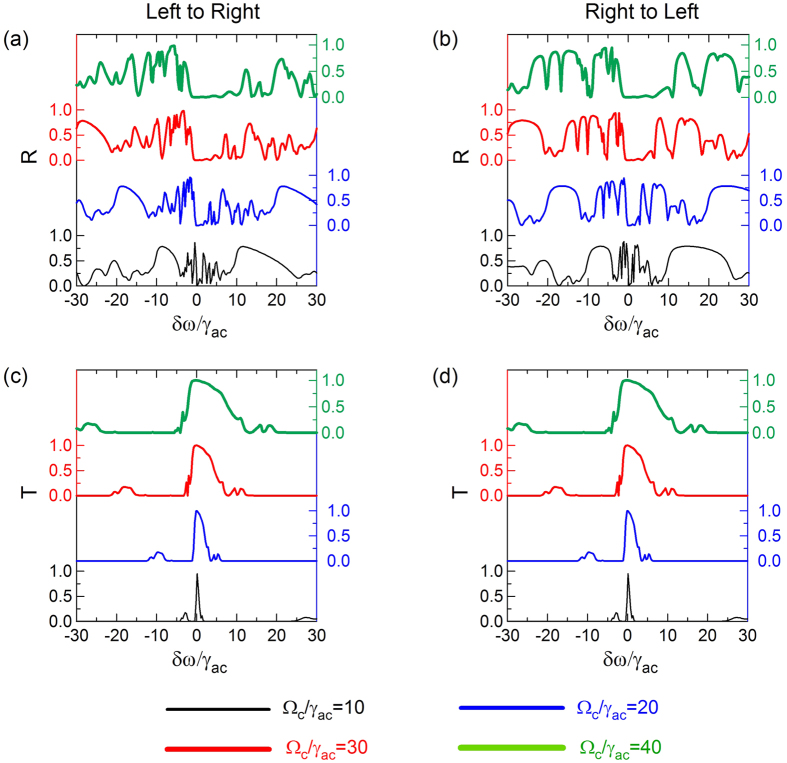
Reflection *R* (**a**,**b**) and transmission *T* (**c**,**d**) of the metamaterial structure shown in Fig. 1 as a function of control beam Rabi frequency Ω_*c*_. Incident probe illuminates the structure from left to right and right to left. The periodic trefoil array is surrounded by vacuum. Ω_*m*_ = 0.

Figure 3a,b present *T* and *R* spectra of the structure as a function of Ω_*c*_ when the probe beam illuminates the structure from left to right (L-R) and right to left (R-L), respectively. Here we assume that the periodic trefoil array is surrounded by air. Figure 3 shows that there are two qualitatively different spectral domains: one where transmission approaches unity (zero reflection) at small *δω*, and one where transmission is significantly lower (large reflection) at large *δω*. Notably, the transmission bandwidth can be tuned by changing the intensity of the control beam, with a wider transmission window for larger control beam intensity. In the center region of high transmission, the phaseonium optical response is dielectric (see for instance the case of Ω_*c*_/*γ*_*ac*_ = 40 in Fig. 3 and the corresponding dielectric functions in Fig. 2b, where *Real* (*ε*) > 0 for *δω*/*γ*_*ac*_ > −5), while in the lossy regions where *Real* (*ε*) < 0 (−40 < *δω*/*γ*_*ac*_ < −5), phaseonium is metallic. Nevertheless, even in the metallic regime the phaseonium metamaterial displays a secondary transmission peak at negative detuning, whose frequency depends on the control beam intensity.

**Figure 4:**
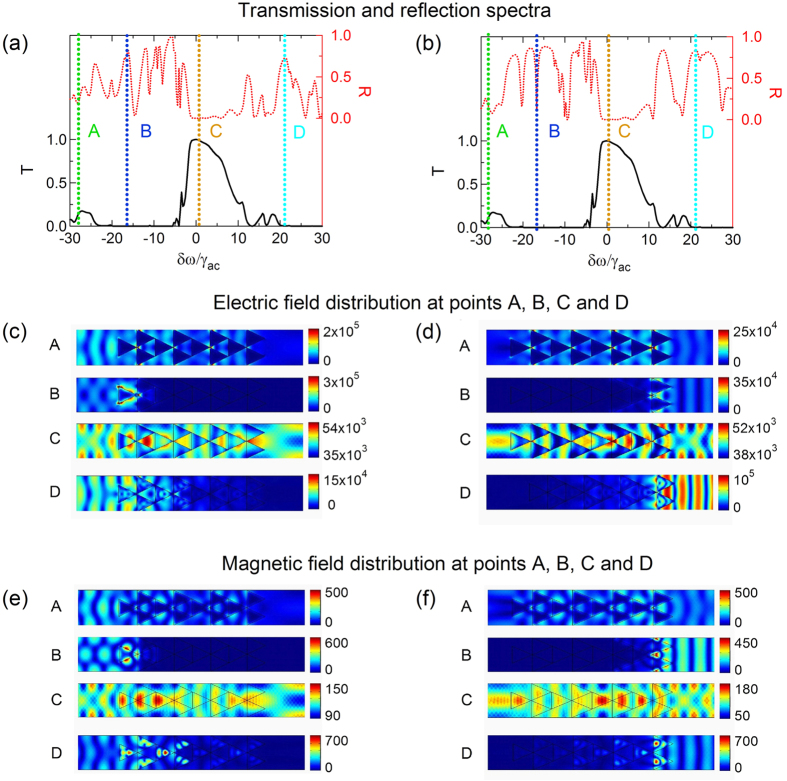
Optical response of the phaseonium metamaterial without microwave excitation (Ω_*m*_ = 0) and Ω_*c*_/*γ*_*ac*_ = 40 (same as in Fig. 3). (**a**,**b**) Transmission and reflection spectra; (**c**,**d**) Electric and (**e**,**f**) magnetic field distributions for selected probe frequencies of *δω* = −27.59 (A, secondary transmission peak), *δω* = −15.23 (B, zero transmission, low reflection), *δω* = 0.75 (C, primary transmission peak) and *δω* = 21.58 (D, zero transmission, high reflection). The incident probe illuminates the structure from left to right (**a**,**c**,**e**) or from right to left (**b**,**d**,**f**).

To further understand the origin of the primary and secondary transmission peaks, we present in Fig. 4 the magnetic and electric field distributions at representative detuning frequencies of *δω* = −27.59 (A, secondary transmission peak), *δω* = −15.23 (B, zero transmission, low reflection), *δω* = 0.75 (C, primary transmission peak) and *δω* = 21.58 (D, zero transmission, high reflection), while the control Rabi frequency was set to Ω_*c*_/*γ*_*ac*_ = 40. In the detuning range −40 < *δω*/*γ*_*ac*_ < −5, the real part of the permittivity is negative, and the phaseonium behaves like a metal. The structure would then support localized surface plasmon polaritons on the corrugated edges of the triangular metamolecules. A closer look to the field distributions reveals that very strong electric fields are trapped at the vertices of the triangles, strongly limiting light transmission. Interestingly, when the detuning achieves phase matching conditions, such as at frequency *δω* = −27.59 (A), coupled surface plasmons can be excited between different metamolecules, thus enabling transmission even when phaseonium is metallic. This explains the existence of the transmission sideband in the metallic regime (see Fig. 3), and the dependence of its center frequency on the control pump Rabi frequency Ω_*c*_, which affects phase matching conditions. At frequency *δω* = −15.23 (B), phase matching conditions are not satisfied, and the transmission vanishes. Here, energy is localized at the vertices of the plasmonic triangular metamolecule closest to the input, creating hot spots. When *δω*/*γ*_*ac*_ > 0, phaseonium is in the dielectric regime and large transmission can be obtained, as seen in the field distributions at frequency *δω* = 0.75 (C). At this frequency, the phaseonium metamaterial can confine and guide light similar to a dielectric waveguide.

Transmission drops drastically at large positive detunings, particularly at *δω* = 21.58 (D) with appearance of reflectance peaks. Typically, in free space, large electric field amplitudes E are accompanied by large magnetic field amplitudes B. However, in the phaseonium structure, fields can be either strongly electric or magnetic, as shown in the field distribution at *δω* = 21.58 (D), where the electric field in the triangle nearest to the incident beam is much stronger than the magnetic field.

As seen in Fig. 3, the transmission spectrum is independent of the direction of the incident probe beam according to the reciprocity principle, while the reflection spectrum shows strong directionality induced by the structure: for instance, at frequency *δω* = −15.23 (B) the R-L reflection is around 80%, while the L-R reflection is only about 20%.

**Figure 5:**
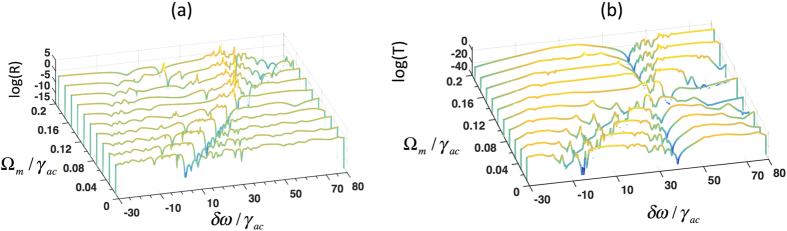
(**a**) Reflection (*R*) and transmission (*T*) of the metamaterial structure as a function of probe frequency detuning *δω* in the presence of microwave Ω_*m*_. The probe light polarization is set to transverse magnetic (TM).

So far, we have discussed the dependence of the reflection and transmission spectra of the phaseonium metamaterial on the control beam Rabi frequency (Ω_*c*_) in the basic scheme without microwave excitation. Figure 2 shows that the permittivity of the three-level system can be strongly modified by a microwave beam. We now show that both amplitude and frequency of transmission or reflection peaks of the phaseonium metamaterial can be controlled actively by changing the microwave Rabi frequency, and that large intensity of the microwave can even induce gain. Figure 5 reports reflection and transmission of the phaseonium metamaterial in semi-logarithmic scale for different microwave Rabi frequencies Ω_*m*_/*γ*_*ac*_ varying from 0.02 to 0.2 while keeping Ω_*c*_/*γ*_*ac*_ = 40 constant. Here the probe light is in transverse magnetic (TM) polarization. When Ω_*m*_ is large enough, starting from Ω_*m*_/*γ*_*ac*_ > 0.005, both transmission and reflection display gain, and the gain peaks blue shifted at larger Ω_*m*_. At large microwave Rabi frequencies, such as Ω_*m*_/*γ*_*ac*_ = 0.2, extremely large gain is observed for both transmission and reflection (see the peaks at *δω*/*γ*_*ac*_ = 50).

**Figure 6:**
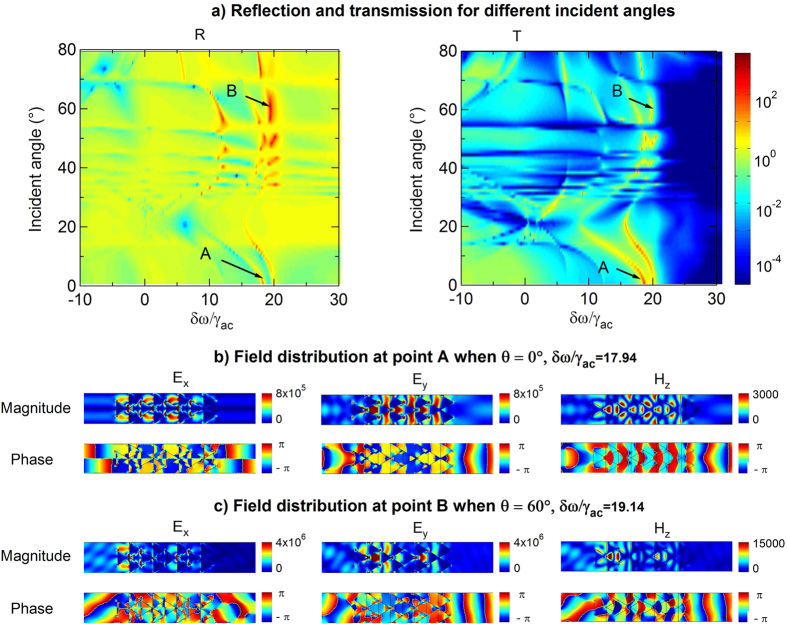
(**a**) Transmission (*T*) and reflection (*R*) spectrum for L-R with silica background. Other parameters are the same as in the previous case of air background. (**b**) Magnetic field distribution at two transmission peaks A and B.

We further studied the optical properties of the metamaterial for finite microwave excitation Ω_*m*_/*γ*_*ac*_ = 0.1, with different incident probe angles *θ* with respect to the *x*-direction, while keeping Ω_*c*_/*γ*_*ac*_ = 40 constant. Figure 6a presents a 2D color map of the reflection and transmission spectra as a function of the probe incident angle. Maximum transmission and reflection peaks are observed at positive detunings *δω*. Although the phaseonium metamaterial can display gain for both transmission and reflection in the presence of the microwave beam (Fig. 5), the transmission acquires higher gain at smaller incidence angle *θ*, while the reflection has larger gain at larger *θ*. This indicates that the system amplifies preferentially the *x*-component of the transmission and the *y*-component of the reflection of the TM-polarized probe beam. Field amplitude distributions and their corresponding phases at maximum transmission [point A in Fig. 6a] and reflection [point B in Fig. 6a] peaks are plotted for two cases, *θ* = 0° and *θ* = 60° in Fig. 6b,c, respectively. In both cases, the electric and magnetic fields are well confined in the *x* − *y* plane within the metamaterial. In this regime, where phaseonium is always metallic (*δω* > −Ω_*c*_), surface plasmon coupling is the main light propagation mechanism. This is evident from the electric and magnetic field distributions, where periodically and highly localized plasmons at the edges of the triangular metamolecules are seen in the high transmission point (Fig. 6b).

Another interesting observation is that, for *θ* = 60°, the phase distributions for the *y*-electric and *z*-magnetic fields evolve from a non-symmetric form along the *y*-direction to a more symmetric form resembling a plane wave, due to the preferential amplification of the transmitted component along the *x*-direction and the resulting change of propagation direction.

**Figure 7:**
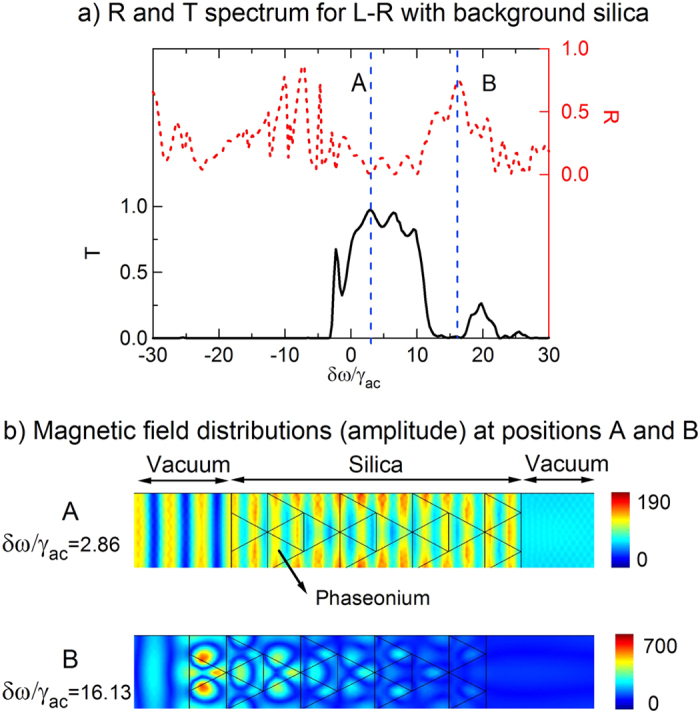
(**a**) Transmission T and reflection R spectrum for L-R with silica background. Other parameters are the same as in the previous case of air background. (**b**) Magnetic field distribution at two transmission peaks A and B.

It has been proposed that phaseonium metamaterial structures could be realized in solid-state systems using patterned crystals doped with rare-earth elements or dielectric matrices embedded with quantum dots^8^,^17^. Since such implementations would require a dielectric matrix to host and hold the phaseonium molecules, we have also investigated the influence of the dielectric background on our phaseonium metamaterial structure. Figure 7 presents the simulations obtained with a silica background (dielectric constant *ε* = 1.5). The change in phase matching condition caused by the silica background leads to the disappearance of the secondary transmission band at negative detuning, observed in the case of air background (Fig. 4), disappear. The magnetic field distribution corresponding to the frequency of nearly perfect transmission (*δω*/*γ*_*ac*_ = 2.86, corresponding to point A in Fig. 7a shows more rapid spatial oscillations. Remarkably, the output magnetic field in this case is nearly homogeneous. Conversely, at the high reflection frequency *δω*/*γ*_*ac*_ = 16.13 (point B in Fig. 7a), the magnetic field steadily weakens throughout the structure, with the strongest field confined at the metamolecule nearest to the input. Compared to the case of air background, here the magnetic field permeates deeper into the phaseonium metamolecules. So, with proper tuning of the coherent control beam parameters, all optical regimes discussed above for phaseonium in air could also be achieved with more realistic dielectric backgrounds.

## Conclusions

In conclusion, we have discussed the structural design of a 2D phaseonium metamaterial consisting of a periodic array of triangular metamolecules arranged in a trefoil. Such metamaterial shows remarkably rich optical properties that can be tuned by the coherent control of laser and maser fields. Depending on the intensity of the maser, the phaseonium optical response ranges from metallic-like to dielectric-like, which allows active tuning of reflection and transmission of the probe beam. At low maser intensities, when phaseonium is predominantly in the dielectric regime, the transmission bandwidth can be varied (from narrow to broad) simply by adjusting the control beam intensity. This can be used for broadband optical switches controlled by amplitude modulation of the laser pump intensity. At high maser intensities, the phaseonium acquires the unique characteristic of a metal with gain. Thanks to the large coupling of surface plasmons, the periodic triangular trefoil structure supports extremely large transmission, with optical amplification. This effect could be exploited to realise optical amplifiers controlled by amplitude modulation of the maser intensity, which would overcome the rapid decay of propagating surface plasmons - the most common problem of lossy plasmonic systems. Furthermore, we showed that preferential amplification of the transmitted component of the probe in the forward direction forces propagation along the metamaterial axis, making this phaseonium metamaterial structure very robust against insertion angle. Finally, we discussed the possible implementation of phaseonium metamaterials in solid-state systems, such as patterned crystals doped with rare-earth elements or dielectric matrices embedded with quantum dots, by analysing the influence of a dielectric background on the optical response. We foresee that the actual implementation of phaseonium metamaterials may result in a new class of active quantum plasmonic devices with unique applications in telecommunications and optical data processing.

## Methods

Optical properties of the quantum coherence material (phaseonium) consisting of an ensemble of of three-level atomic-like systems can be controlled by Rabi frequencies Ω_*c*_ and Ω_*m*_ of the external control light and microwave, respectively. According to calculations in^8^ and^9^, the complex susceptibility of phaseonium can be calculated by


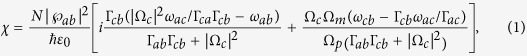


where *ħ* and *ε*_0_ are Planck constant and electric constant respectively. The decoherence functions Γ_*in*_ are given by Γ_*ca*_ = *γ*_*ac*_ + *i*Δ_*c*_, Γ_*ab*_ = *γ*_*ab*_ − *i*Δ(*ω*) and Γ_*cb*_ = *γ*_*bc*_ + *i*(Δ_*c*_ − Δ(*ω*). The effective decay rates *γ*_*in*_ depend on the spontaneous emission rates Γ_*i*_ and defacing rates. In our simulation, we use the following parameters for quantum dot based^8^,^17^: 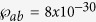 Cm and *N* = 10^24^ m^−3^. Finally, the permittivity of the phaseonium can be calculated by


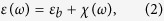


where *ε*_*b*_ is the dielectric constant of the background medium which is equal to 1 for air and 1.5 for fused silica.

The transmission and reflection of the phaseonium structure with different probe direction and corresponding electric and magnetic field distributions, were computed by the finite element method (FEM) in the COMSOL package, using the refractive index dispersion of phaseonium deduced from Eq. 2. Floquet boundary condition was used to mimic the infinitely periodic structure in the *y*-direction.

## Additional Information

**How to cite this article**: Nguyen, D. M. *et al.* Coherently Tunable Triangular Trefoil Phaseonium Metamaterial. *Sci. Rep.* **6**, 21083; doi: 10.1038/srep21083 (2016).
